# Supplementary material for: Network enhancement as a general method to denoise weighted biological networks
Source: Nat Commun. 2018 Aug 6;9:3108. doi: 10.1038/s41467-018-05469-x (PMC6078978; doi:10.1038/s41467-018-05469-x)
Supplement: Supplementary file 1 — Supplementary Information [file 41467_2018_5469_MOESM1_ESM.pdf]

Network Enhancement: a general method to denoise weighted biological networks

Supplementary information

Bo Wang,[1,*] Armin Pourshafeie,[2,*] Marinka Zitnik,[1,*] Junjie Zhu,[3]
Carlos D. Bustamante,[4,5] Serafim Batzoglou,[1,‡,#] and Jure Leskovec[1,5,#]

[1] Department of Computer Science, Stanford University, Stanford, CA, USA
[2] Department of Physics, Stanford University, Stanford, CA, USA
[3] Department of Electrical Engineering, Stanford University, Stanford, CA, USA
[4] Department of Biomedical Data Science, Stanford University, Stanford, CA, USA
[5] Chan Zuckerberg Biohub, San Francisco, CA, USA
[‡] Currently at Illumina Inc.

[*] These authors contributed equally.

[#] To whom correspondence should be addressed. E-mail: serafim@cs.stanford.edu, jure@cs.stanford.edu

Supplementary Figures

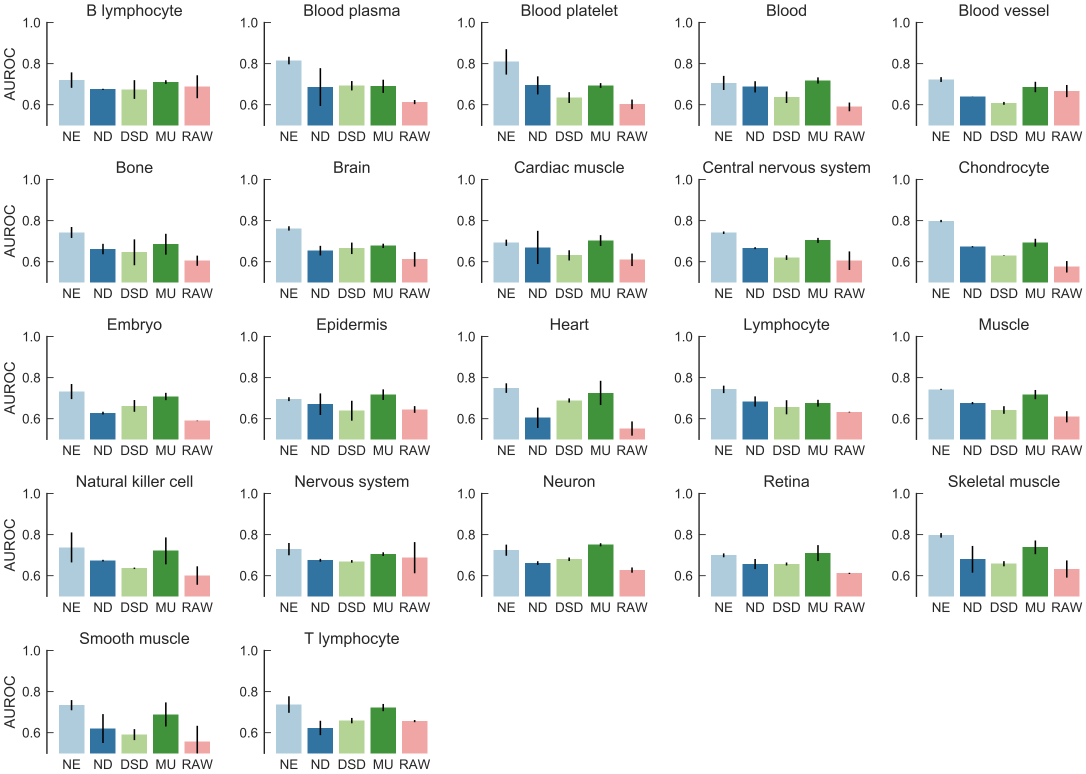

Supplementary Figure 1: **Gene function prediction for genome-wide gene interaction networks in human tissues using a leave-one-out cross-validation setting.** We considered tissue-specific gene interaction networks [1] (RAW) and their denoised versions, which we obtained by applying MU, ND, DSD or NE to the original (RAW) networks. We then used the networks to predict gene functions specific to each tissue as defined in Greene *et al.* [1]. Each bar indicates the performance of a random-walk based approach that was applied to a raw or a denoised network in order to predict gene functions taking place in the tissue described by the network. Prediction performance is measured using AUROC, where a high AUROC value indicates the approach successfully learned to rank an actual gene-function association higher than a random gene-function pair. Error bars indicate performance variation across all gene functions in a given tissue. Results are shown for all 22 human tissues considered in this study. The average AUROC values achieved by the methods across 22 tissues are: NE: 0.742, ND: 0.662, DSD: 0.649, MU: 0.706, and RAW: 0.616.

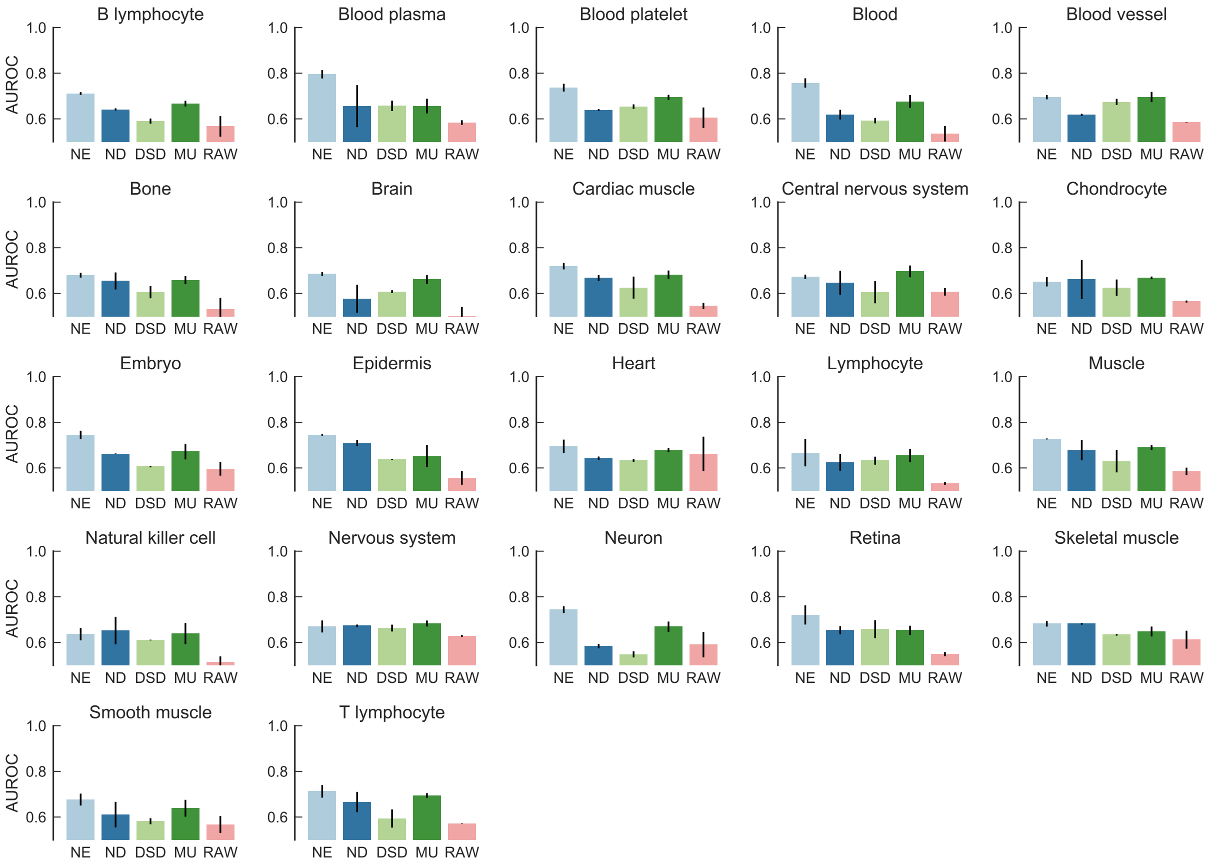

Supplementary Figure 2: **Gene function prediction for genome-wide gene interaction networks in human tissues using a 5-fold cross-validation setting.** We considered tissue-specific gene interaction networks [1] (RAW) and their denoised versions, which we obtained by applying MU, ND, DSD or NE to the original (RAW) networks. We then used the networks to predict gene functions specific to each tissue as defined in Greene *et al.* [1]. Each bar indicates the performance of a random-walk based approach that was applied to a raw or a denoised network in order to predict gene functions taking place in the tissue described by the network. Prediction performance is measured using AUROC, where a high AUROC value indicates the approach successfully learned to rank an actual gene-function association higher than a random gene-function pair. Error bars indicate performance variation across all gene functions in a given tissue. Results are shown for all 22 human tissues considered in this study. The average AUROC values achieved by the methods across 22 tissues are: NE: 0.706, ND: 0.646, DSD: 0.621, MU: 0.669, and RAW: 0.572.

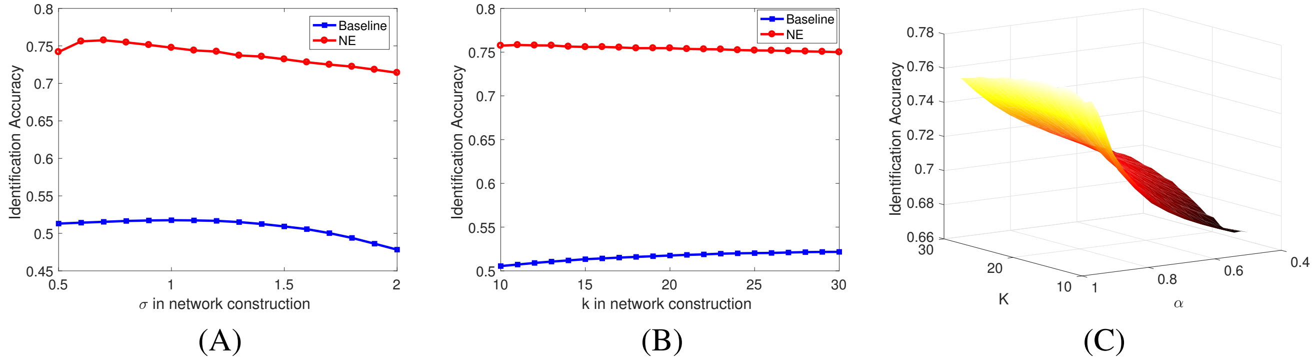

Supplementary Figure 3: **Results of NE on fine-grained species identification.** (**A**) shows the sensitivity to the hyper-parameter, σ, when constructing the similarity network on butterfly dataset. (**B**) shows the sensitivity to the hyper-parameter k for this network. (**C**) is a mesh plot of different values of K and α for our network enhancement on the butterfly dataset.

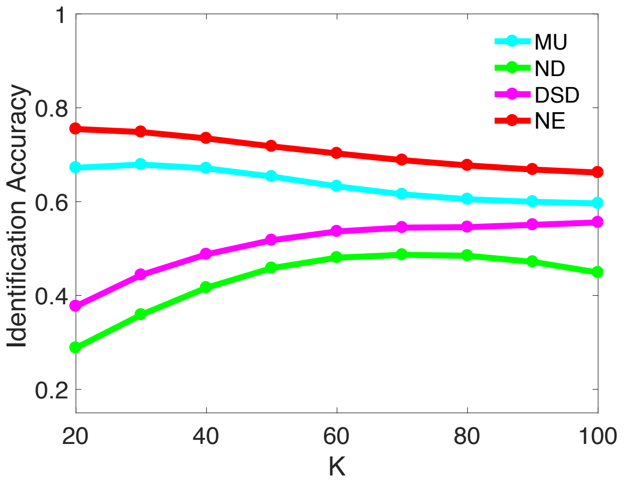

Supplementary Figure 4: **Species Identification Accuracy with respect to different number of K in K-NN pruning as a pre-processing step.** We perform the same KNN pruning for all methods and report the corresponding identification accuracy. It is observed that, NE outperforms the alternative methods for various choices of K. Furthermore, both NE and MU perform better for smaller K, the performance of DSD improves as K is increased and ND performs best at an intermediate value in the range investigated.

Supplementary Note 1: Further Information on Datasets

Tissue-Specific Gene Interaction Networks

Tissue-specific gene interaction networks were retrieved from the GIANT (Genome-scale Integrated Analysis of gene Networks in Tissues) database [1]: http://giant.princeton.edu. Networks were filtered to only include edges with evidence supporting a tissue-specific functional interaction (*i.e.,* network type "top edges" in the GIANT database). Each network was used as input to a network denoising algorithm to clean the network edges. The resulting denoised network was then used as input to a random-walk based algorithm to predict gene functions.

Gene functions were defined by the Gene Ontology (GO) terms [2]. Gene-function associations were specified by the GO annotations [2] and retrieved from ftp://ftp.ncbi.nlm.nih.gov/gene/DATA/gene2go.gz in August 2016. We only used high confidence annotations associated with the experimental evidence codes: EXP, IDA, IMP, IGI, IEP, ISS, ISA, ISM or ISO, and further removed all annotations with a non-empty "qualifier" column [3]. The original GO files only contained the most specific annotations explicitly. We therefore added all implicit more general annotations by up-propagating the given annotations along the full GO tree.

We obtained the mapping of GO terms to tissues, that is, associations between tissues and tissue-specific functions, from Greene *et al.* [1]. Greene *et al.* used text matching followed by manual curation to map GO terms to tissues. GO terms were filtered to only include those with at least 20 associated genes. As a result, there were 22 tissues with each having at least one tissue-specific gene function. In total, there were 309 tissue-specific gene functions across all 22 tissues. Tissues with the largest number of functions were: natural killer cell (49 GO terms), lymphocyte (43 GO terms) and muscle (34 GO terms).

To predict gene functions we used a random-walk based approach. Random walks were used before to transfer GO annotations within networks ([4–8] and many others) and were shown to be among the top-perfoming approaches for gene function prediction [5,6]. We defined a random walk starting from nodes that were known to be associated with a query gene function and were included in the training set. At each time step, the walk had a probability r of returning to the initial nodes. We set $r = 0.75$, as was done by Köhler *et al.* [4]. Once the random walk process converged (L2-distance between probability vectors in consecutive time steps $< 10^{-6}$), predictions were made for all nodes in the test set based on their visitation probability. Predictions were evaluated against known gene-function associations using a leave-one-out cross-validation strategy.

Hi-C Interaction Networks

For each autosome, the provided contact matrix (counts per bin) from Rao et al. [9] was normalized using SQRTVC as defined in [9]. In their work Rao et al. also introduced the Arrowhead algorithm as a way of detecting clusters within a Hi-C adjacency matrix. The Arrowhead algorithm produces

clusters that may overlap. Since the true clusters are unknown, to generate a confident set of labels, for each chromosome, we sub-sampled the first 15 non-overlapping clusters that contain no sub-clusters as determined by the arrowhead algorithm [9]. We chose non-overlapping clusters as both of the community detection algorithm we use for post processing are limited to detecting non-overlapping communities [10,11]. This sub-sampled adjacency matrix constitutes the contact matrix for our new Hi-C interaction network. For visualization purposes, we only show the first 9 communities. We have chosen chromosome 16 as our visualization example. This example was chosen to have a performance just below the median as measured by NMI of Louvian clustering for 1kb resolution Hi-C.

Fine-Grained Image Datasets and Similarity Networks

First, we test our method on a dataset with 10 different classes of butterflies, each of which containing 55 to 100 images totaling to 832 butterflies [12]. We use two different encoding methods (Fisher Vector (FV) [13,14] and Vector of Linearly Aggregated Descriptors (VLAD) [15] with dense SIFT [16]) to generate two different descriptors for these images. These two encoding methods describe the statistics of the codebooks differently and therefore we use our method to combine them.

Given a feature set that describes a collection of images, denoted as $X = \{x_1, x_2, \ldots, x_n\}$, we want to construct a similarity graph $\mathcal{N} \in R^{n\times n}$ in which $\mathcal{W}(i,j)$ indicates the kernel value between the i-th and j-th object. The most widely used method assumes a Gaussian distribution across pairwise similarities:

$$\mathcal{W}(i,j) = \exp\left(-\frac{\|x_i - x_j\|^2}{2\sigma^2}\right).$$

Here, σ is a hyper-parameter that needs careful manual tuning. To overcome the sensitivity to σ, a more advanced method of constructing similarity kernels is proposed in [17] where the variance is estimated using the local scales of the distances as follows. Assume k is the number of neighbors. For each cell, e.g, x_i, the associated local variance is estimated as:

$$\epsilon_i = \frac{\sum_{j\in\mathcal{KNN}(i)} \|x_i - x_j\|}{k},$$

where $\mathcal{KNN}(i)$ denotes all the top k neighbors of the i-th cell. Thus the new kernel is defined as:

$$\mathcal{W}_k^\sigma(i,j) = \exp\left(-\frac{\|x_i - x_j\|^2}{\sigma^2(\epsilon_i + \epsilon_j)^2}\right).$$

We set $k = 20$ and $\sigma = 0.5$ as default values.

Supplementary Note 2: Definition of Evaluation Metrics

Normalized Mutual Information

Throughout the paper, we used Normalized Mutual Information (NMI) [18] to evaluate the consistency between the obtained clustering and the true labels of the cells. Given two clustering results U and V on a set of data points, NMI is defined as: $I(U,V)/\max\{H(U),H(V)\}$, where $I(U,V)$ is the mutual information between U and V, and $H(U)$ represents the entropy of the clustering U.

Specifically, assuming that U has P clusters, and V has Q clusters, the mutual information is computed as follows:

$$I(U,V) = \sum_{p=1}^{P}\sum_{q=1}^{Q}\frac{|U_p \cap V_q|}{N}\log\frac{|U_p \cap V_q|}{|U_p|/N \times |V_q|/N},$$

where N is the number of points and $|U_p|$ denotes the cardinality of the p-th cluster in U. The entropy of each cluster assignment is calculated as follows:

$$H(U) = -\sum_{p=1}^{P}\frac{|U_p|}{N}\log\frac{|U_p|}{N},$$

$$H(V) = -\sum_{q=1}^{Q}\frac{|V_q|}{N}\log\frac{|V_q|}{N}.$$

Further details on NMI can be found in Vinh et al.[19]. NMI takes on values between 0 and 1 where a higher NMI indicates a higher concordance between the two sets, *i.e.,* a more consistent label assignment.

Retrieval Accuracy

We use retrieval accuracy for evaluation of fine-grained image retrieval. For a single query q, the accuracy on k retrievals is defined as:

$$acc(q,k) = \frac{\text{\# of correct retrievals}}{min(k, N_q)},$$

where N_q is the number of objects with the same label of q. Here, "correct retrievals" mean the retrieved images from the same class of q. We also report the mean accuracy Acc over all the images in the dataset:

$$Acc = \frac{1}{n}\sum_{i=1}^{n} acc(q_i, N_{q_i}),$$

where n is the number of images in the dataset.

Supplementary Note 3: Theoretical Analysis of Network Enhancement

Doubly Stochastic Matrix

Here, we state the definition of a Doubly Stochastic Matrix (*DSM*):

Definition 1. Given a matrix $M \in \mathbb{R}^{n\times n}$, M is a *Doubly Stochastic Matrix (DSM)* if it satisfies the following two conditions:

1. $M_{i,j} \geq 0 \quad i, j \in \{1, 2, \ldots, n\}$,
2. $\sum_i M_{i,j} = \sum_j M_{i,j} = 1$.

Remark. The largest eigenvalue of a DSM matrix is 1. It is easy to check that $\mathbf{1} = (1, 1, \ldots 1)^T$ is a right eigenvector with eigenvalue 1. Similarly, $\mathbf{1}^T$ is a left eigenvector with eigenvalue 1. For an irreducible DSM, Perron-Frobenius theorem implies that the $(\mathbf{1}, 1)$ pair is unique and 1 is the largest eigenvalue. When M is reducible, its indices can be split to construct k irreducible DSM's. Any eigenvector of M needs to be an eigenvector of all of these matrices. Since the eigenvalue corresponding to each of these matrices cannot be greater than 1 we conclude that the largest eigenvalue of a reducible DSM is 1 corresponding to eigenvector $\mathbf{1}$ and potentially other eigenvectors.

Next, we show that the transition matrix is a DSM. First we re-state the construction of transition matrices:

$$P_{i,j} \leftarrow \frac{W_{i,j}}{\sum_{k\in\mathcal{N}_i} W_{i,k}} * I\{j \in \mathcal{N}_i\}, \qquad \mathcal{T}_{i,j} \leftarrow \sum_{k=1}^{n} \frac{P_{i,k}P_{j,k}}{\sum_{v=1}^{n} P_{v,k}}. \tag{1}$$

Where $I\{\cdot\}$ is an indicator function. By checking the conditions from the definition of DSM, we verify that $\mathcal{T}$ is a symmetric DSM.

Given a weighted graph $W \in \mathbb{R}^{n\times n}$, the transition probability matrix $P = D^{-1}W$, where D is the diagonal matrix whose entries are the degree of the vertices, i.e., $D_{ii} = \sum_{j=1}^{n} W_{i,j}$. In other words, we have:

$$P_{i,j} = \frac{W_{i,j}}{\sum_{k=1}^{n} W_{i,k}}. \tag{2}$$

It is easy to verify that, $P\mathbb{1} = \mathbb{1}$, *i.e.,* the row sum of P is always 1. Note P is not symmetric. Now we construct the DSM matrix $\mathcal{T}$ as follows:

$$\mathcal{T}_{i,j} \leftarrow \sum_{k=1}^{n} \frac{P_{i,k}P_{j,k}}{\sum_{v=1}^{n} P_{v,k}}. \tag{3}$$

It is easy to see that, $\mathcal{T} \in \mathbb{R}^{n\times n}$ is symmetric:

$$\mathcal{T}_{i,j} = \sum_{k=1}^{n} \frac{P_{i,k}P_{j,k}}{\sum_{v=1}^{n} P_{v,k}} = \sum_{k=1}^{n} \frac{P_{j,k}P_{i,k}}{\sum_{v=1}^{n} P_{v,k}} = \mathcal{T}_{j,i}.$$

It remains to show that $\mathcal{T}$ is a DSM.

Since weights are assumed to be non-negative, $W_{i,j} \geq 0$. This implies that $P_{i,j}$ as defined in equation 2 is non-negative and therefore, $\mathcal{T}_{i,j} \geq 0$

Next we show that the second property of DSM holds by by first proving $\mathcal{T}\mathbf{1} = \mathbf{1}$:

$$(\mathcal{T}\mathbf{1})_i = \sum_{j=1}^{n}\sum_{k=1}^{n} \frac{P_{i,k}P_{j,k}}{\sum_{v=1}^{n} P_{v,k}} = \sum_{k=1}^{n} P_{i,k} \frac{\sum_{j=1}^{n} P_{j,k}}{\sum_{v=1}^{n} P_{v,k}} = \sum_{k=1}^{n} P_{i,k} = 1. \tag{4}$$

This implies that, each row sum of $\mathcal{T}$ is 1, so $\mathcal{T}\mathbf{1} = \mathbf{1}$. If we take transpose on both sides, we have $\mathbf{1}'\mathcal{T}' = \mathbf{1}'$, and since T is symmetric (i.e., $\mathcal{T}' = \mathcal{T}$), then we obtain $\mathbf{1}'\mathcal{T} = \mathbf{1}'$. So, we conclude that the row sums and the column sums of $\mathcal{T}$ are always 1. This proves that $\mathcal{T}$ is a DSM. Put together, we have that $\mathcal{T}$ is symmetric doubly stochastic matrix.

Further, we can see that $\mathcal{T}$ will be positive semi-definite. To show this, for any vector $\mathbf{z}$, we need to prove $\mathbf{z}'\mathcal{T}\mathbf{z} \geq 0$:

$$\begin{aligned}
\mathbf{z}'\mathcal{T}\mathbf{z} &= \sum_{i=1}^{n}\sum_{j=1}^{n} z_i z_j \mathcal{T}_{i,j} = \sum_{i=1}^{n}\sum_{j=1}^{n} z_i z_j \sum_{k=1}^{n} \frac{P_{i,k}P_{j,k}}{\sum_{v=1}^{n} P_{v,k}} \\
&= \sum_{k=1}^{n} \frac{\sum_{i=1}^{n}\sum_{j=1}^{n} z_i z_j P_{i,k}P_{j,k}}{\sum_{v=1}^{n} P_{v,k}} = \sum_{k=1}^{n} \frac{(\sum_{i=1}^{n} z_i P_{i,k})^2}{\sum_{v=1}^{n} P_{v,k}} \geq 0.
\end{aligned}$$

We thus confirmed that $\mathcal{T}$ is positive semi-definite.

Furthermore, we can easily verify that convex combinations of symmetric DSMs is still a symmetric DSM.

Proof. This proof follows immediately from the definition. Given m DSMs, A_i, for $i = 1, 2, \ldots, m$, a convex combination is $\sum_i^m \beta_i A_i$, such that $\sum_i^m \beta_i = 1$ and $\beta_i \geq 0, i = 1, \ldots, m$. The symmetry of a convex combination of symmetric matrices is trivial. The first property of DSM follows since all values involved are non-negative and are added or multiplied. The second property is also easy to confirm using $(\sum_i \beta_i A_i)\mathbf{1} = \sum_i \beta_i (A_i \mathbf{1}) = \mathbf{1}$. Transposing this equation and using symmetry shows the results for the column sums. □

Network Enhancement Preserves Properties of DSM

Network enhancement diffusion process is given by:

$$W_{t+1} = \alpha\mathcal{T} \times W_t \times \mathcal{T} + (1-\alpha)\mathcal{T}, \tag{5}$$

where initialization is done by $W_{t=0} \leftarrow W$, with α a regularization parameter, and t representing the iteration number.

Theorem 1. *In each iteration t of network enhancement (NE) as defined by Eqn. (5), the following properties hold :*

1. *W_t remains a symmetric DSM.*
2. *W_t converges to a non-trivial equilibrium graph that is a symmetric DSM.*
3. *W_t remains positive-semi definite if $W_{t=0}$ is positive semi-definite.*

Proof. To prove the first statement, we focus on checking the definitions. Given that $W_{t=0}$ and the local graph $\mathcal{T}$ are symmetric DSMs, we can proceed by induction on t. Assume W_t is a symmetric DSM, we want to verify that W_{t+1} is again symmetric and a DSM. We start by proving symmetry:

$$W'_{t+1} = \alpha(\mathcal{T}W_t\mathcal{T})' + (1-\alpha)\mathcal{T} = \alpha(\mathcal{T}'W'_t\mathcal{T}') + (1-\alpha)\mathcal{T} = \alpha(\mathcal{T}W_t\mathcal{T}) + (1-\alpha)\mathcal{T} = W_{t+1}.$$

Here, we use $W'_t = W_t$ and $\mathcal{T}' = \mathcal{T}$. Hence, W_{t+1} is symmetric.

We proceed to show that W_{t+1} remains doubly stochastic. It is obvious that each element of W_{t+1} is non-negative. To show the rows and columns remain normalized, we note that:

$$W_{t+1}\mathbf{1} = \alpha\mathcal{T}W_t\mathcal{T}\mathbf{1} + (1-\alpha)\mathcal{T}\mathbf{1} = \alpha\mathcal{T}W_t\mathbf{1} + (1-\alpha)\mathcal{T}\mathbf{1} = \alpha\mathcal{T}\mathbf{1} + (1-\alpha)\mathcal{T}\mathbf{1} = \mathcal{T}\mathbf{1} = \mathbf{1}.$$

here we have used $\mathcal{T}\mathbf{1} = \mathbf{1}$ and $W_t\mathbf{1} = \mathbf{1}$, since they are both DSMs. This shows that W_{t+1} is row normalized. We can appeal to symmetry to show that the matrix will also be column normalized which shows statement 1.

Next we show that it is possible to find a closed form solution for the final, converged network. We start by first providing an expression for the network at iteration t. Then we find the network in the limit of large number of iterations.

Define $W_0 = W_{t=0}$. For iteration t, the following holds true:

$$W_t = \alpha^t\mathcal{T}^tW_0\mathcal{T}^t + (1-\alpha)\mathcal{T}\sum_{k=0}^{t-1}(\alpha\mathcal{T}^2)^k. \tag{6}$$

which can be shown by induction. For $t=1$, $W_{t=1} = \alpha\mathcal{T}W_0\mathcal{T} + (1-\alpha)\mathcal{T}$, and clearly satisfies Eqn. (6). Assume Eqn. (6) is true for iteration t. Then:

$$\begin{aligned} W_{t+1} &= \alpha\mathcal{T}W_t\mathcal{T} + (1-\alpha)\mathcal{T} \\ &= \alpha\mathcal{T}(\alpha^t\mathcal{T}^tW_0\mathcal{T}^t + (1-\alpha)\mathcal{T}\sum_{k=0}^{t-1}(\alpha\mathcal{T}^2)^k)\mathcal{T} + (1-\alpha)\mathcal{T} \\ &= \alpha^{t+1}\mathcal{T}^{t+1}W_0\mathcal{T}^{t+1} + (1-\alpha)\mathcal{T}\sum_{k=0}^{t}(\alpha\mathcal{T}^2)^k. \end{aligned}$$

which satisfies Eqn. (6). Let $t \to \infty$, then:

$$W_{t\to\infty} = (1-\alpha)\mathcal{T}(\mathcal{I} - \alpha\mathcal{T}^2)^{-1}.$$

This proves that the network enhancement process converges to a non-trivial equilibrium graph $W_{t\to\infty} = (1-\alpha)\mathcal{T}(\mathcal{I}-\alpha\mathcal{T}^2)^{-1}$. Note that this result is the limit of symmetric DSM matrices. The set of symmetric $n \times n$ doubly stochastic matrices can be described by $\{M : M - M^T = 0, M_{i,j} \geq 0, \sum_i M_{i,j} = 1, \sum_j M_{i,j} = 1\}$. Since these conditions are inverse images of closed sets ($\{0\}, [0,\infty), \{1\}, \{1\}$ respectively) under continuous maps, the set of symmetric DSMs is closed and contains the limit point corresponding to the converged diffusion network in NE.

Lastly, we argue that if W_0 is positive semi-definite, then the NE diffusion process preserves this property at every iteration. By induction, let W_t be positive semi-definite then for any vector $\mathbf{z} \in \mathbb{R}^n$:

$$\mathbf{z}'W_{t+1}\mathbf{z} = \alpha\mathbf{z}'\mathcal{T}W_t\mathcal{T}\mathbf{z} + (1-\alpha)\mathbf{z}'\mathcal{T}\mathbf{z} = \alpha(\mathcal{T}\mathbf{z})'W_t(\mathcal{T}\mathbf{z}) + (1-\alpha)\mathbf{z}'\mathcal{T}\mathbf{z} \geq 0.$$

Finally, we argue that since the set of positive semi-definite matrices can be represented by $\{M : f(M) \geq 0\}$ where $f(M) = \min_{\|x\|=1}\langle x, Mx\rangle$ is a continuous function, the set of positive semi-definite matrices is closed (and thus contains it's limit points) as it is the inverse image of $[0,\infty)$ under f. □

This theorem demonstrates that the diffusion process in NE preserves some important properties of the original network. Importantly, at every stage of the diffusion process, the results corresponds to an undirected network with the same normalization as the initial network.

Spectral Analysis of Network Enhancement

Now we present our main novel finding that the proposed network enhancement process does not change eigenvectors of the initial symmetric DSM while mapping eigenvalues via a non-linear function.

Theorem 2. *Let $(\lambda_0, \mathbf{v}_0)$ denote the eigen-pair of a symmetric DSM $\mathcal{T}_0$. Then the network enhancement process defined in Eqn. (5) does not change the eigenvectors and the final converged graph has an eigen-pair $(f_\alpha(\lambda_0), \mathbf{v}_0)$, where $f_\alpha(x) = \frac{(1-\alpha)x}{1-\alpha x^2}$.*

Proof. Let $\mathcal{T}_0$ denote the initial symmetric DSM and $\mathcal{T}_\infty$ denote the final symmetric DSM. From the proof above, it is easy to see that the final network $\mathcal{T}_\infty$ is given by $\mathcal{T}_\infty = (1-\alpha)\mathcal{T}_0(\mathcal{I}-\alpha\mathcal{T}_0^2)^{-1}$. Since $\mathcal{T}_0$ is a symmetric DSM, then we have $\mathcal{T}_0 = U\Sigma U^{-1}$ where U is the set of eigenvectors and

Σ is a diagonal matrix whose entries are eigenvalues of $\mathcal{T}_0$, i.e., $\Sigma_{i,i} = \lambda_i$. Clearly,

$$\begin{aligned}
\mathcal{T}_\infty &= (1-\alpha)\mathcal{T}_0(\mathcal{I} - \alpha\mathcal{T}_0^2)^{-1} \\
&= (1-\alpha)U\Sigma U^{-1}(\mathcal{I} - \alpha U\Sigma U^{-1}U\Sigma U^{-1})^{-1}. \\
&= (1-\alpha)U\Sigma U^{-1}(UU^{-1} - \alpha U\Sigma U^{-1}U\Sigma U^{-1})^{-1}. \\
&= (1-\alpha)U(\Sigma(\mathcal{I} - \alpha\Sigma^2)^{-1})U^{-1}.
\end{aligned}$$

Hence, we obtain the eigen-decompostion of $\mathcal{T}_\infty$. That is, the eigenvectors are still U but the eigenvalues becomes $\Sigma'_{i,i} = (1-\alpha)\lambda_i(1-\alpha\lambda_i^2)^{-1}$. This completes the proof of the theorem. □

This theorem shows that, the defined network enhancement process using a DSM is a nonlinear operator on the eigenvalue-spectrum of the network. This theorem not only provides us with a closed-form expression for obtaining the final network at convergence but also sheds light on how network enhancement process improves the graph. First, if the original eigenvalues are either 0 or 1, the network enhancement process preserves these eigenvalues. Second, network enhancement process always decreases the eigenvalues since $\frac{(1-\alpha)\lambda_0}{1-\alpha\lambda_0^2} \leq \lambda_0$. More importantly, NE increases the eigengaps between large eigenvalues (Lemma 1) and thereby enhances the robustness of the obtained graph (Theorem 3) and influences clustering. Third, while all eigenvalues are reduced, the non-linear function f_α reduces small eigenvalues more aggressively than large eigenvalues. In this sense, NE acts similar to a smoothed out version of PCA but does not completely diminish any singular value.

Consider the initial graph $\mathcal{T}_0 \in \mathbb{R}^{n\times n}$ and the obtained graph $\mathcal{T}_\infty \in \mathbb{R}^{n\times n}$ after the network enhancement process. Then,

Lemma 1. *Let,* $c(\alpha) = \sqrt{-\frac{\sqrt{\alpha^2-10\alpha+9}+\alpha-3}{2\alpha}}$*, for all eigenvectors with eigengap contained in* $[1, c(\alpha)]$ *(i.e.* $\lambda_{i+1} \geq c(\alpha)$*) the eigengap is larger in* $\mathcal{T}_\infty$ *than in* $\mathcal{T}_0$*.*

Proof. First we note that by Theorem 2, $\mathcal{T}_\infty, \mathcal{T}_0$ share the same eigenvectors. Let k be the last eigenvector with $\lambda_{k+1} \geq c(\alpha)$. The lemma reduces to showing:

$$\|\lambda_j - \lambda_{j+1}\| \leq \|\lambda_j^{(\infty)} - \lambda_{j+1}^{(\infty)}\|, \text{ with } j \leq k$$

where $\lambda_j^{(\infty)}$ is the j-th eigenvalues of the final graph. By Theorem 2, we have $\lambda_j^{(\infty)} = \frac{(1-\alpha)\lambda_j}{1-\alpha\lambda_j^2}$, therefore, the preceding equations becomes:

$$\lambda_j - \frac{(1-\alpha)\lambda_j}{1-\alpha\lambda_j^2} \leq \lambda_{j+1} - \frac{(1-\alpha)\lambda_{j+1}}{1-\alpha\lambda_{j+1}^2}.$$

Since $\lambda_j \geq \lambda_{j+1}$, the claim holds where $g_\alpha(x) = x - \frac{(1-\alpha)x}{1-\alpha x^2}$ is a decreasing function. Differentiating $g_\alpha(x)$, gives the following condition:

$$\frac{\partial g_\alpha(x)}{\partial x} = 1 - (1-\alpha)\frac{(1+\alpha x^2)}{(1-\alpha x^2)^2} \leq 0.$$

Since $0 < \alpha < 1$, this condition implies that: $x^4\alpha + x^2(\alpha - 3) + 1 \geq 0$, or that:

$$|x| \geq \sqrt{-\frac{\sqrt{\alpha^2 - 10\alpha + 9} + \alpha - 3}{2\alpha}} = c(\alpha).$$

□

One implication of this lemma is an increased robustness. For $H \in \mathbb{R}^{n\times n}$, a symmetric perturbation, define $\overline{M} := M + H$ as the perturbed version of M. Further, denote the eigenspace spanned by the largest k eigenvectors of M by $V_{M,k}$. Then, let $\mathbf{dist}(V_M, V_{\overline{M}})$ indicates the distance between projected eigenspaces of M and $\overline{M}$ (see detailed definition in the review by Von Luxburg [20]).

Theorem 3. *(Perturbation Analysis) $\mathcal{T}_\infty$ has a better resistance to noise than $\mathcal{T}_0$ in the following sense:*

$$\sup_{\substack{\|H\|=h \\ \mathcal{T}_0}} \{\mathbf{dist}(V_{\mathcal{T}_0,k}, V_{\overline{\mathcal{T}_0},k})\} \geq \sup_{\substack{\|H\|=h \\ \mathcal{T}_0}} \{\mathbf{dist}(V_{\mathcal{T}_\infty,k}, V_{\overline{\mathcal{T}_\infty},k})\},$$

for all k with $\lambda_{k+1} \geq c(\alpha)$ in $\mathcal{T}_0$.

To prove this theorem, the key observation lies in the fact that for large eigenvalues, the eigengap of $\mathcal{T}_0$ is always smaller than the corresponding eigengap of $\mathcal{T}_\infty$.

Proof. First, we directly use a modified version of Davis-Kahan theorem (Theorem 2 from [21]). The text of theorem 2 from [21] is reproduced below for completeness:

Let $\Sigma, \hat{\Sigma} \in \mathbb{R}^{p\times p}$ be symmetric, with eigenvalues $\lambda_1 \geq \cdots \geq \lambda_p$ and $\hat{\lambda}_1 \geq \cdots \geq \hat{\lambda}_p$ respectively. Fix $1 \leq r \leq s \leq p$ and assume that $\min(\lambda_{r-1} - \lambda_r, \lambda_s - \lambda_{s+1} > 0)$ where $\lambda_0 := \infty$ and $\lambda_{p+1} := -\infty$. Let $d := s - r + 1$, and let $V = (v_r, v_r + 1, \ldots, v_s) \in \mathbb{R}^{p\times d}$ and $\hat{V} = (\hat{v}_r, \hat{v}_{r+1}, \ldots, \hat{v}_s) \in \mathbb{R}^{p\times d}$ have orthogonal columns satisfying $\Sigma v_j = \lambda_j v_j$ and $\hat{\Sigma}\hat{v}_j = \hat{\lambda}_j \hat{v}_j$ for $j = r, r+1, \ldots, s$. Then:

$$\|\sin\Theta(\hat{V}, V)\|_\mathrm{F} \leq \frac{2\min(d^{1/2}\|\hat{\Sigma} - \Sigma\|_\mathrm{op}), \|\hat{\Sigma} - \Sigma\|_\mathrm{F})}{\min(\lambda_{r-1} - \lambda_r, \lambda_s - \lambda_{s+1})}.$$

Moreover, there exists an orthogonal matrix $\hat{O} \in \mathbb{R}^{d\times d}$ such that:

$$\|\hat{V}\hat{O} - V\|_\mathrm{F} \leq \frac{2^{3/2}\min(d^{1/2}\|\hat{\Sigma} - \Sigma\|_\mathrm{op}, \|\hat{\Sigma} - \Sigma\|_\mathrm{F})}{\min(\lambda_{r-1} - \lambda_r, \lambda_s - \lambda_{s+1})}.$$

Here, $\|sin(\Theta(V_1, \hat{V}_1))\|$ is the angle between subspaces V_1 and $\hat{V}_1$.

Let $\lambda_i^{(\infty)}$ denote the ith eigenvalue of $\mathcal{T}_\infty$ and $\lambda_i^{(0)}$ denote the ith eigenvalue of $\mathcal{T}_0$. Then from Lemma 1, we see that any $\mathcal{T}_\infty$ corresponds to a $\mathcal{T}_0$ with a smaller eigengap between eigenvalues $(\lambda_i - \lambda_{i+1})$ for $i \geq k$ Then, using the theorem above, we can conclude that for $i \leq k$, the upper bound is smaller for $\mathcal{T}_\infty$ than for $\mathcal{T}_0$. Since this upper bound is sharp [21], this proves the theorem.

□

Remark 1. Note that Theorem 3 holds true for any $V = \text{span}(\lambda_i, \ldots, \lambda_{i+j})$ for $i + j \leq k, j \geq 0$, that is, the subspace spanned by any range of $(j + 1)$ "large" eigenvalues.

Here, the focus on large eigenvalues is particularly relevant for the problem of community detection. To see this, consider an undirected network with k connected components. Such a network can be represented as a block-diagonal, symmetric DSM with k degenerate eigenvalues equal to 1. In a real-world setting, there may be true edges that violate the block-diagonal structure. If we treat these edges as small perturbations over the block-diagonal matrix, by Weyl's inequality, we expect the eigenvalues of the perturbed matrix to remain close to those of the original (block-diagonal) matrix. i.e., the eigenvalues remain close to 1.

Remark 2. Lemma 1 provides insight about the role of α. Recall that $c(\alpha)$ is an increasing function of α. In our experiments we have used $\alpha = 0.85$ corresponding to $c(\alpha) = 0.78$. Since the eigenvalues are restricted to stay within the $[0, 1]$ interval and to preserve their signs, the algorithm compresses the gap between small eigenvalues (i.e., eigenvalues below $c(\alpha)$) in order to expand the gap between large eigenvalues (i.e., eigenvalues above $c(\alpha)$). We make the following three observations: α controls: (1) which interval will go through compression and which interval will go through expansion, (2) the intensity of this compression/expansion, and (3) the non-linearity of this compression/expansion.

Supplementary Figure 3C empirically shows that the results of NE are not sensitive to the value of α. This stability is due to the relative flatness of $c(\alpha)$, $c(0.15) = 0.6$, $c(0.85) = 0.78$, indicating that the expansion region is not very sensitive to the value of α away from the extreme ends. At the end points of α, $c(\alpha)$ changes rapidly. For example, when $\alpha = 1$ the algorithm reduces to a simple diffusion algorithm (without a restart). In that case, the expansion region is only $\{1\}$ and all other eigenvalues are compressed to $\{0\}$ as is expected in a pure diffusion algorithm.

Supplementary References

1. Greene, C. S. *et al.* Understanding multicellular function and disease with human tissue-specific networks. *Nature Genetics* **47**, 569–576 (2015).

2. Ashburner, M. *et al.* Gene Ontology: tool for the unification of biology. *Nature Genetics* **25**, 25–29 (2000).

3. Berriz, G. F., Beaver, J. E., Cenik, C., Tasan, M. & Roth, F. P. Next generation software for functional trend analysis. *Bioinformatics* **25**, 3043–3044 (2009).

4. Köhler, S., Bauer, S., Horn, D. & Robinson, P. N. Walking the interactome for prioritization of candidate disease genes. *The American Journal of Human Genetics* 949–958 (2008).

5. Navlakha, S. & Kingsford, C. The power of protein interaction networks for associating genes with diseases. *Bioinformatics* **26**, 1057–1063 (2010).

6. Radivojac, P., Clark, W. T., Oron, T. R. *et al.* A large-scale evaluation of computational protein function prediction. *Nature Methods* **10**, 221–7 (2013).

7. Yu, G., Zhu, H., Domeniconi, C. & Liu, J. Predicting protein function via downward random walks on a gene ontology. *BMC Bioinformatics* **16**, 271 (2015).

8. Wang, S., Cho, H., Zhai, C., Berger, B. & Peng, J. Exploiting ontology graph for predicting sparsely annotated gene function. *Bioinformatics* **31**, i357–i364 (2015).

9. Rao, S. S. *et al.* A 3d map of the human genome at kilobase resolution reveals principles of chromatin looping. *Cell* **159**, 1665–1680 (2014).

10. Blondel, V. D., Guillaume, J.-L., Lambiotte, R. & Lefebvre, E. Fast unfolding of communities in large networks. *Journal of Statistical Mechanics: Theory and Experiment* **10**, 10008 (2008). 0803.0476.

11. Le Martelot, E. & Hankin, C. Fast Multi-Scale Community Detection based on Local Criteria within a Multi-Threaded Algorithm. *ArXiv e-prints* (2013). 1301.0955.

12. Wang, J., Markert, K. & Everingham, M. Learning models for object recognition from natural language descriptions. In *BMVC* (2009).

13. Perronnin, F., Sánchez, J. & Mensink, T. Improving the fisher kernel for large-scale image classification. In *ECCV* (2010).

14. Jorge Sánchez, F. P. & Akata, Z. Fisher vectors for fine-grained visual categorization. In *CVPR* (2011). URL http://hal.archives-ouvertes.fr/docs/00/81/76/81/PDF/fgvc11.pdf.

15. Jégou, H., Douze, M., Schmid, C. & Pérez, P. Aggregating local descriptors into a compact image representation. In *CVPR*, 3304–3311 (IEEE, 2010).

16. Bosch, A., Zisserman, A. & Muoz, X. Image classification using random forests and ferns. In *ICCV*, 1–8 (2007).

17. Wang, B. *et al.* Similarity network fusion for aggregating data types on a genomic scale. *Nature Methods* **11**, 333–337 (2014).

18. Strehl, A. & Ghosh, J. Cluster ensembles—a knowledge reuse framework for combining multiple partitions. *The Journal of Machine Learning Research* **3**, 583–617 (2003).

19. Vinh, N. X., Epps, J. & Bailey, J. Information theoretic measures for clusterings comparison: Variants, properties, normalization and correction for chance. *Journal of Machine Learning Research* **11**, 2837–2854 (2010). URL http://dl.acm.org/citation.cfm?id=1756006.1953024.

20. Von Luxburg, U. A tutorial on spectral clustering. *Statistics and Computing* **17**, 395–416 (2007).

21. Yu, Y., Wang, T. & Samworth, R. J. A useful variant of the davis–kahan theorem for statisticians. *Biometrika* **102**, 315–323 (2014).
